# Characterization of the complete chloroplast genome of *Taxus wallichiana* as the medicinal plant from China

**DOI:** 10.1080/23802359.2020.1799725

**Published:** 2020-11-11

**Authors:** Tian Ruan, Ruijing Tan, WenJie Ma, Minghang Li, Xuyi Zheng, Lifen He

**Affiliations:** a Second Clinical Medical School of Kunming Medical University, Kunming, Yunnan, China; b Kunming Medical University, Kunming, Yunnan, China; c First Clinical Medical School of Kunming Medical University, Kunming, Yunnan, China

**Keywords:** *Taxus wallichiana*, Taxaceae, chloroplast genome, phylogenetic relationship

## Abstract

*Taxus wallichiana* is a member of the family Taxaceae, which is a unique and endangered species in China and is widely used for ornamental, material and medicinal purposes. The complete chloroplast genome of *T. wallichiana* was found to possess a total size of 128,168 bp. The GC content of *T. wallichiana* chloroplast genome sequence is 37.3%, the overall nucleotide composition of chloroplast genome sequence is: A of 30.7%, T of 32.0%, C of 19.0% and G of 18.3%. The total of 116 genes were successfully annotated, which contained 83 protein-coding genes, 29 transfer RNA genes, and 4 ribosomal RNA genes. The ML phylogenetic analysis result showed that *T. wallichiana* was closely related to *Taxus baccata* in the phylogenetic relationship using the neighbour-joining (NJ) method in this study.

*Taxus wallichiana* is the IUCN Red List species in the world, which is also one of the most important tree and medicinal plant species in China. *Taxus wallichiana* is one of the slow growing family Taxaceae species that is found to be the major source of Taxol used as the anti-cancer agent (Sudina and Dhurva 2018). Taxol is originally isolated from the bark of *T. wallichiana*, which is one of the most effective antitumor drugs for the treatment of several cancers, such as breast, lung and ovarian cancers (Croteau et al. 2006). Currently, very less is known about the biology and genomics information of *T. wallichiana*. In this paper, the complete chloroplast genome of *T. wallichiana* was characterized and generated that can be used for genome information resource collection and further drug development research for this species.

*Taxus wallichiana* fresh samples were collected from the Kunming Medical University, which were located at 102.83E, 24.85 N (Kunming, Yunnan, China) and stored at the −80 °C refrigerator future use. The *Taxus wallichiana* genomic DNA was extracted from the fresh leaves using the modified CTAB method, which was stored in Kunming Medical University (No.KMMU-001) and sequenced. Furthermore, the reads quality were controlled and removed using the FastQC (Andrews 2015). The chloroplast genome sequence of *T. wallichiana* was employed to obtain and assemble using the MitoZ (Meng et al. 2019). The chloroplast genome annotation was conducted using Geneious (Kearse et al. 2012). All the coding and other genes were predicted using the CPGAVAS (Liu et al. 2012) for this chloroplast genome. At last, the circular chloroplast genome map of *T. wallichiana* was generated using OrganellarGenomeDRAW online (Greiner et al. 2019).

The complete chloroplast genome of *T. wallichiana* (NCBI No.NK9584421) was found to possess a total size of 128,168 bp. The GC content of *T. wallichiana* chloroplast genome sequence is 37.3%, the overall nucleotide composition of chloroplast genome sequence is: A of 30.7%, T of 32.0%, C of 19.0% and G of 18.3%. The total of 116 genes were successfully annotated, which contained 83 protein-coding genes, 29 transfer RNA genes, and 4 ribosomal RNA genes.

To ascertain the position of *T. wallichiana* within other plant species, the maximum-likelihood (ML) phylogenetic tree was reconstructed using 13 species complete chloroplast genome sequence from NCBI (Figure 1). In this paper, the ML phylogenetic tree was reconstructed using PhyML (Guindon et al. 2010) with 200 bootstrap replicates and with GTR + G + I as the best-fit nucleotide substitution model. At last, the ML phylogenetic tree was edited using MEGA X (Kumar et al. 2018). The ML phylogenetic analysis result showed that *T. wallichiana* was closely related to *Taxus baccata* in the phylogenetic relationship in this study. The result can be used for the use of active ingredients of traditional Chinese medicine and drug development research in future.

**Figure 1.**
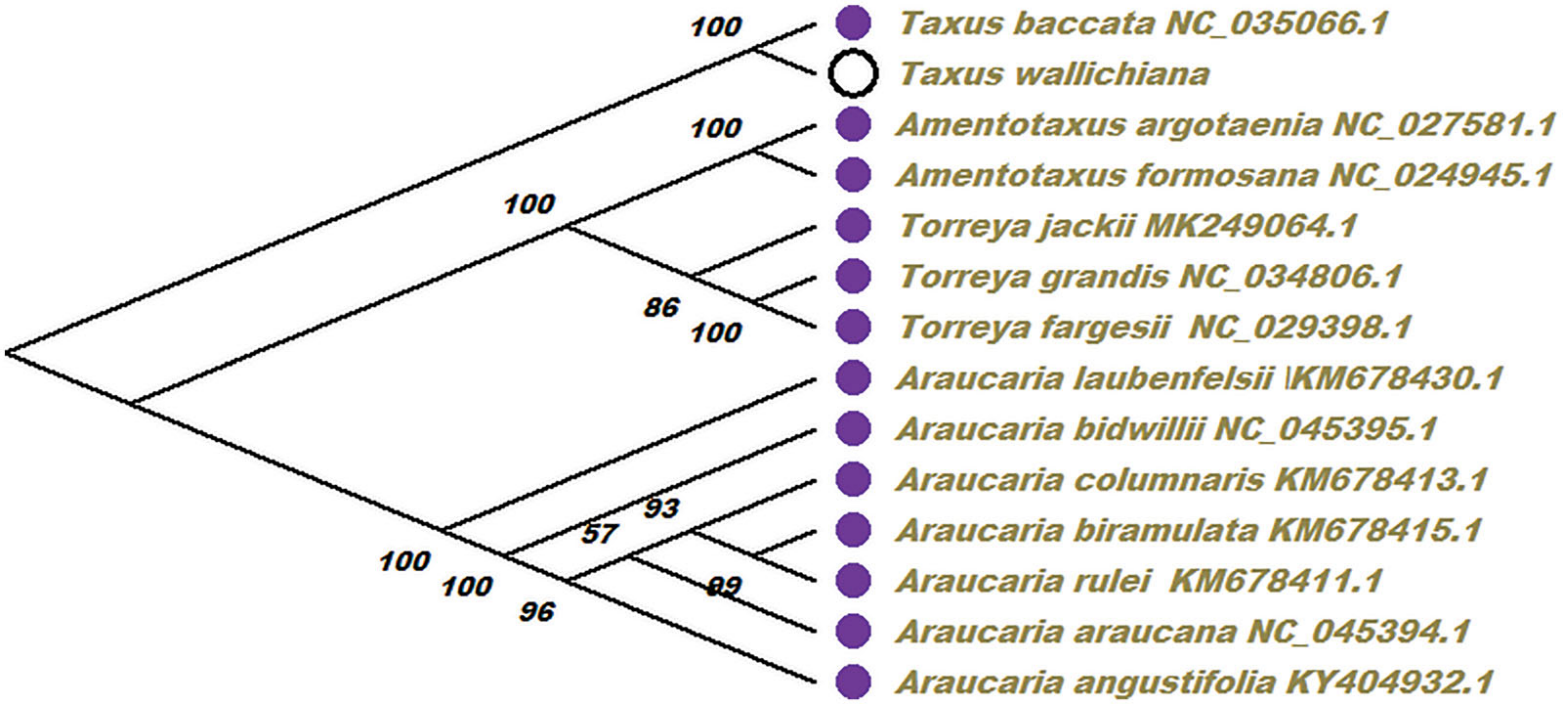
Phylogenetic relationships of 13 species within *T. wallichiana* based on the maximum-likelihood (ML) analysis chloroplast genome sequence from NCBI. The bootstrap values were based on 2000 replicates, and are shown next to the branches.

## Data Availability

The data that support the findings of this study are available from the corresponding author, upon reasonable request. The data that support the findings of this study are openly available in *Taxus wallichiana* at NCBI and http://doi.org/10.1080/23802359.2020.1799725, reference number [reference number NK9584421].
